# Defense Priming and Jasmonates: A Role for Free Fatty Acids in Insect Elicitor-Induced Long Distance Signaling

**DOI:** 10.3390/plants5010005

**Published:** 2016-01-08

**Authors:** Ting Li, Tristan Cofer, Marie Engelberth, Jurgen Engelberth

**Affiliations:** 1 Department of Biology, University of Texas at San Antonio, One UTSA Circle, San Antonio, TX 78249, USA; tuy746@my.utsa.edu (T.L.); marie.engelberth@utsa.edu (M.E.); 2 Environmental Science Academic Program, University of Texas at San Antonio, One UTSA Circle, San Antonio, TX 78249, USA; uew998@my.utsa.edu

**Keywords:** jasmonic acid, green leaf volatiles, free fatty acids, linolenic acid, palmitoleic acid, plant volatiles, signaling, insect elicitor

## Abstract

Green leaf volatiles (GLV) prime plants against insect herbivore attack resulting in stronger and faster signaling by jasmonic acid (JA). In maize this response is specifically linked to insect elicitor (IE)-induced signaling processes, which cause JA accumulation not only around the damage site, but also in distant tissues, presumably through the activation of electrical signals. Here, we present additional data further characterizing these distal signaling events in maize. Also, we describe how exposure to GLV increases free fatty acid (fFA) levels in maize seedlings, but also in other plants, and how increased fFA levels affect IE-induced JA accumulation. Increased fFA, in particular α-linolenic acid (LnA), caused a significant increase in JA accumulation after IE treatment, while JA induced by mechanical wounding (MW) alone was not affected. We also identified treatments that significantly decreased certain fFA level including simulated wind and rain. In such treated plants, IE-induced JA accumulation was significantly reduced when compared to un-moved control plants, while MW-induced JA accumulation was not significantly affected. Since only IE-induced JA accumulation was altered by changes in the fFA composition, we conclude that changing levels of fFA affect primarily IE-induced signaling processes rather than serving as a substrate for JA.

## 1. Introduction

Plants in their natural or agricultural habitats are constantly exposed to a plethora of pest and pathogens. Among the pests, insect herbivores have adapted over 350 million years of co-evolution to identify appropriate host plants for feeding and oviposition [1]. Therefore, the survival of plants strongly depends on their ability to respond effectively to this threat [1]. Aside from constitutive defenses like mechanical barriers or stored toxic products, plants have also developed a wide array of inducible defenses. Typical inducible defense responses to insect herbivory include the production of proteins that block digestion, toxic secondary metabolites, and the release of volatile organic compounds (VOC) [1,2]. Most of these countermeasures are initiated by the induction of jasmonic acid (JA) [2]. JA is synthesized through the octadecanoid signaling pathway, starting with α-linolenic acid (LnA). Two organelles participate in JA biosynthesis: the chloroplast and the peroxisome. While the first steps, including the oxygenation by a lipoxygenase (for maize LOX8) and cyclization, take place in the chloroplast and result in the formation of 12-oxo-phytodienoic acid, subsequent steps, including the reduction of the cyclopentenone and three rounds of β-oxidation pathway, occur in the peroxisome and complete the conversion into JA [2]. JA then needs to be conjugated to an amino acid—for example isoleucine—to become bioactive [2,3]. The induction of this pathway in response to insect herbivory can be activated by damage (or mechanical wounding (MW)) alone, or by a combination of damage and the simultaneous application of insect-derived elicitors (IE) abundant in the oral secretions of the herbivores [4,5,6,7,8,9,10,11,12,13,14].

In some plants, mechanical wounding (MW) alone is sufficient to induce JA accumulation both locally at the wounding site and systemically in undamaged tissue [4,5,6,7,8,9]. In tomato and other solanaceous plants, systemic signaling after MW is mediated by systemin, an 18-amino acid peptide derived from prosystemin. Systemin is produced by wounded leaf cells and travels to companion cells where it binds to a receptor. This triggers a signaling cascade that results in the accumulation of JA, which is then transported to other tissues [4,5,6,7]. In *Arabidopsis*, MW also leads to the accumulation of JA-isoleucine (JA-Ile) locally and systemically within minutes after treatment [8]. It was further shown that the systemic accumulation was not due to transport of JA or JA-Ile from the damage site, but *de-novo* synthesis [8]. Recently, it was demonstrated that so-called “system potentials” may be responsible for long distance signaling after wounding and insect herbivory in *Vicia faba* and *Hordeum vulgare* [9]. These system potentials, which are based on hyperpolarization of the plasma membrane (PM) through the activation of the PM H^+^-ATPase, travel at a speed of 5–10 cm·min^−1^ through the plant, and may very well be responsible for the activation of defense responses in systemic tissues [9]. In *Arabidopsis*, system potentials were also found to be activated after MW [10]. Moreover, mutations in glutamate receptor-like proteins attenuated these surface potentials resulting in significantly reduced JA-dependent gene expression in systemic tissues, strongly suggesting that glutamate receptor-like proteins play an important role in the generation of long distance signals [10]. Interestingly, while hyperpolarization is responsible for long distance wound signaling in several plant species, tomato plants responded to hyperpolarizaion by fusicoccin—an activator of the PM H^+^-ATPase—by repressing typical wound genes, and instead activated the expression of pathogenesis-related proteins [11]. But while for some plants MW is sufficient to induce long-distance defense signaling, other plants, including maize and tobacco, require the presence of IE at the damage site for this to occur [12,13,14].

IE (also referred to as herbivore-associated molecular patterns or HAMPS) have been shown to induce defense responses in maize, and other plants, that were very similar to those observed after actual insect herbivory [12,13,14]. A class of very potent IE are amino acid-conjugates of fatty acids [15,16], in particular volicitin and *N*-linolenoyl-glutamine. Other IE that have been identified in recent years were the inceptins, a peptide elicitor isolated from *Spodoptera frugiperda*, and the califerins from the grasshopper *Schistocerca americana* [17,18]. In a study by Schmelz *et al.* [19] it was shown that in maize, volicitin and linolenoyl-glutamine had the highest biological activity when measured as induced JA accumulation.

In maize, MW alone induced JA accumulation only at the damage site. In contrast, IE induced JA accumulation not only around the damaged site, but also in distal (leaf upwards) tissues [12,13]. Moreover, expression analyses revealed that the bulk of defense gene expression also occurred in those areas with increased JA accumulation [13]. Only MYC7—a putative ortholog of the *Arabidopsis* MYC2 transcription factor, which plays an important role in mediating JA responses—was found in basal parts of the treated leaf as well as in systemic leaves [13].

IE were also characterized by their ability to induce the production of volatiles in plants, which were often very similar to those induced by actual herbivory. These VOC, which mainly consist of products of the shikimic acid pathway, terpenes, and fatty acid-derived products like green leaf volatiles (GLV) [20,21], have been shown to be a very effective countermeasure by repelling further infestation [22] and attracting predators and parasites of the attacking herbivore [23,24,25,26]. Among those VOC emitted after insect herbivore damage GLV are of particular interest. GLV were first characterized at the beginning of the last century [27], but were considered as shunt metabolites from the pathway leading to traumatin: the first wound hormone described for plants [28]. The biosynthetic pathway leading to the production of GLV is well understood [27,28,29,30,31,32,33]. GLV are fatty acid-derived products formed from LnA and linoleic acid, which serve as substrates for a pathway-specific 13-lipoxygenase (for maize LOX10 [31]). The resulting 13-hydroperoxy LnA is then cleaved by the enzyme hydroperoxide lyase (HPL) producing *Z*-3-hexenal (from 18:3 fatty acids) or hexanal (from 18:2 fatty acids) as well as 12-oxo-(*Z*)-9-decenoic acid. Further processing of *Z*-3-hexenal by alcohol dehydrogenase, acetylation and isomerization leads to the production of the remaining C_6_-components, like *Z*-3-hexenol, *Z*-3-hexenyl acetate, and the respective *E*-2-enantiomers. GLV are almost immediately released locally after wounding (20) with *Z*-3-hexenal peaking within the first 2–3 min, while *Z*-3-hexenol and *Z*-3-hexenyl acetate are produced delayed at lower rates [33]. Recent research showed that while *Z*-3-hexenal is biosynthesized by the damaged tissue, *Z*-3-hexenol and *Z*-3-hexenyl acetate are produced by neighboring intact cells [33]. Besides their association with vegetative tissue GLV are also major constituents of volatiles emitted by flowers and fruits.

While GLV may also play important roles in the immediate wound response, investigations into their activity as volatile defense signals between plants have become a major focus of research in the last decade. In 2004 it was found that maize seedlings exposed to GLV rapidly accumulate JA and emit small amounts of VOC [34]. Moreover, maize seedlings exposed overnight to GLVs from neighboring plants produced significantly more JA and VOC compared to their controls when treated with IE. This was the first report on priming against insect herbivory signaled by GLV and we found that this effect is specifically linked to defense response. This priming response induced by GLV has since been confirmed for several other plant species including lima beans, wild tobacco, poplar, and *Arabidopsis*, and appears to be common to all plants [35,36,37,38,39].

While other volatile compounds, such as *cis* jasmone, methyl jasmonate, and several terpenes, have also been described to induce defense related genes [40,41,42], none of them exhibited any priming-related activity among different plant species as it has been shown for GLV. However, recently indole has been described as another volatile priming signal [43], but its effect on plants other than maize has yet to be established. Also, while the composition of VOC released in response to herbivore damage varies significantly among different species—in particular with regard to the above-mentioned active compounds—all plants investigated so far release GLV when mechanically damaged indicating they are universal signaling compounds [29,30].

Priming plant defense responses resulting in an accelerated and/or enhanced reaction is well established [44] and usually works through one or more of the commonly-studied defense signaling pathways (SA-, JA-, ethylene-mediated). However, it is still unclear how priming of herbivore-specific defense responses by GLV is regulated. While in some plants exposure to GLV causes the accumulation of JA—in particular in monocots—other plants (mainly dicots) do not show this response. Therefore, JA does not seem to be the common factor that regulates defense priming against insect herbivores. The goal of this study was to further characterize the response of maize to insect elicitor and establish a correlation with GLV-induced primed responses. At the center of this study is our finding that changes in free fatty acid (fFA) levels—in particular increases in LnA—emerge as a common response of plants to treatments with GLV. Furthermore, we show that these changes have a significant and specific stimulatory effect on the response of maize plants to insect elicitors. Also, we show that movement of plants results in significantly reduced LnA levels, and that these lower levels correlate with reduced JA accumulation after IE treatment. The specific modulation of IE-induced JA accumulation by fFA may be caused by altered long distance signaling processes by a yet to be characterized mechanism.

## 2. Results and Discussion

### 2.1. Priming by Green Leaf Volatiles Affect Local and Distal Responses to Insect Elicitors

**Figure 1:**
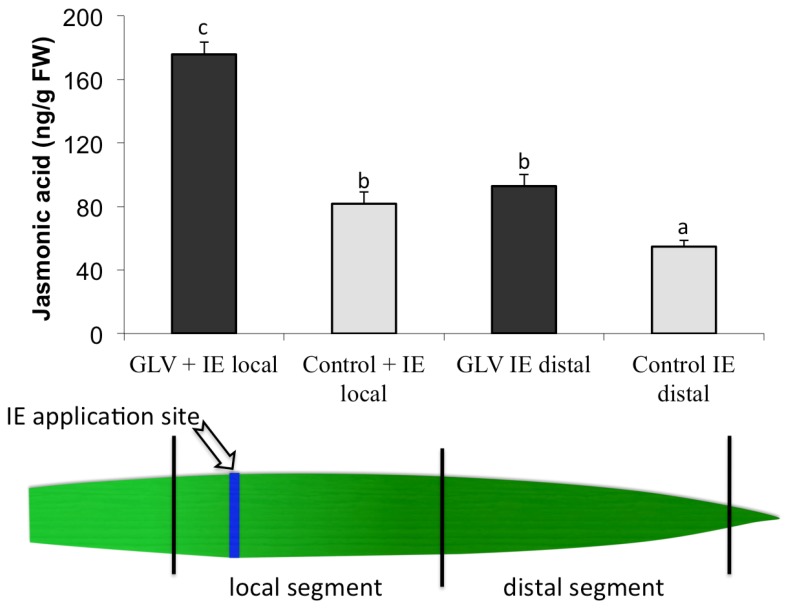
Effect of *Z*-3-hexenol (GLV) priming on local and distal jasmonic acid (ng·gFW^−1^) accumulation after treatment with insect elicitor (IE). Plants were exposed to 10 μg of GLV for 16 h and then IE for 1 h. Controls were treated similarly, but without GLV. Jasmonic acid was analyzed by GC/MS as described in [19,34,45]. Different letters above each bar indicate statistical difference determined by ANOVA analysis followed by Tukey tests where appropriate (*p* < 0.05). *N* = 4, error bars represent standard deviation.

Previously, we showed that priming of plant defense responses by green leaf volatiles (GLV) resulted in increased jasmonic acid (JA) accumulation and volatile production in maize seedlings treated with insect elicitor (IE) when compared to non-primed controls [34]. Moreover, we showed that the distribution of JA accumulation along the IE-treated leaves of GLV primed plants was not restricted to the IE application site, but also occurred in the distal portion of the same leaf [12]. This local/distal distribution of JA accumulation was further reflected in the expression of defense-related genes, while neither JA nor defense gene transcripts accumulated significantly in the basal part of the leaf [13]. To test the effects of GLV-induced defense priming on local/distal JA accumulation we treated maize plants with physiological concentrations of *Z*-3-hexenol for 16 h, and then with IE [34], and analyzed JA levels 60 min later. We found that not only the local area (*i.e.*, the IE application site and 2.5 cm of the distal portion of the leaf), but also the far distal area of the same leaf showed increased JA accumulation when compared to non-primed IE-treated controls (Figure 1).

### 2.2. Insect Elicitor-Induced Signaling Rapidly Travels to Distant Tissues

**Figure 2:**
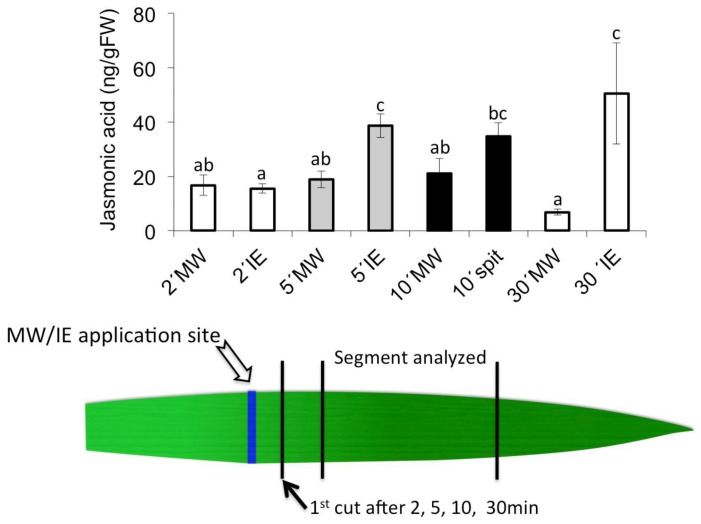
Characterization of distal signaling speed induced by mechanical wounding (MW) and insect elicitors (IE) treatment at 2 min, 5 min, 10 min, and 30 min. Different letters above each bar indicate statistical difference determined by ANOVA analysis followed by Tukey tests where appropriate (*p* < 0.05). *N* = 4, error bars represent standard deviation.

We have shown previously that mechanical wounding (MW) does induce JA accumulation only locally at the damage site, but not in distal or basal sections of the same leaf [12,13]. Also, it was shown that IE bind to the plasma membrane at or around the application site, and no movement of this elicitor through the plant was detected [46]. This strongly suggests that IE-induced signaling events travel quickly towards the distal portion of the leaf. To further characterize signaling events involved in the IE-induced distal accumulation of JA, we tested for the speed of signaling after IE treatment. As a control we treated maize seedlings with MW alone. Selected leaves were treated with IE by scratching an area of approximately 2 mm × 10 mm on the second leaf of intact maize plants with a razor blade and 10 μL of IE solution were immediately added to the wounded site. After 2 min, 5 min, 10 min, and 30 min intervals, individual leaves were taken and cut 1 cm above the IE-application site. Cut leaves were then placed in 4 mL vials with water and allowed to rest for a total of 45 min. After cutting another 1 cm from the base of the leaf to eliminate the damage site of the first cut, we analyzed JA accumulation in the remaining distal part of the leaf. While JA did not accumulate above control levels in this distal section when the IE-application site was cut-off after 2 min, we found the full induction of JA in distal tissues that were cut after 5 min (Figure 2). No further changes in maximum JA accumulation were detected in those leaves that were cut at 10 and 30 min after IE application. At the same time, mechanical wounding (MW) alone did not induce any JA accumulation confirming our previous results regarding the lack of activity of MW in distal tissues.

### 2.3. Jasmonic Acid Is Not a Mobile Signal

**Figure 3:**
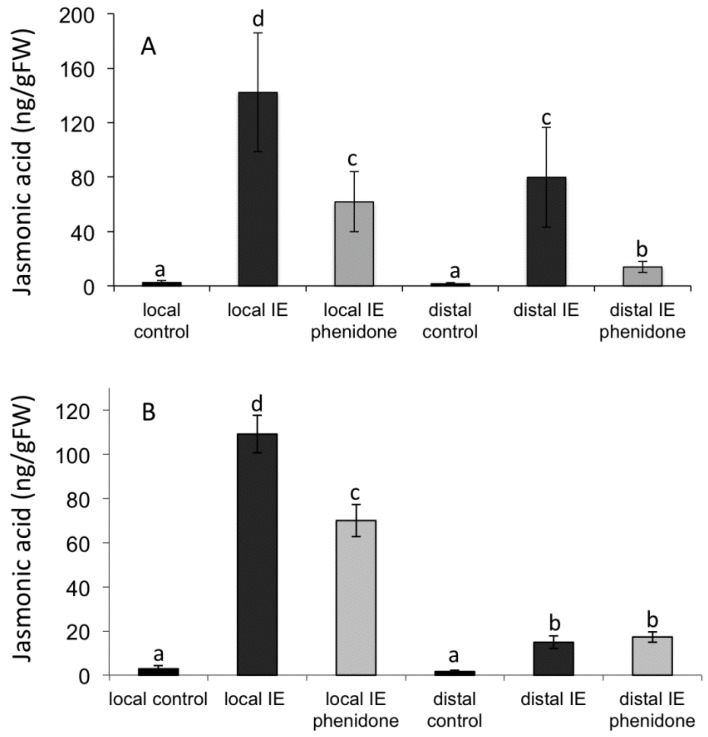
(**A**) Effect of cut stem application of phenidone on local and distal signaling after treatment with insect elicitor (IE); (**B**) Effect of local application of phenidone on local and distal signaling after treatment with IE. Different letters above each bar indicate statistical difference determined by ANOVA analysis followed by Tukey tests where appropriate (*p* < 0.05). *N* = 3, error bars represent standard deviation.

The speed of this distal signaling, however, does not allow for any conclusions about the nature of the mobile signal. Phloem transport of signaling molecules—in particular JA and other oxylipins—might be one option as measured transport speeds of other solutes were found to be more than sufficient to cover distances of 1 cm well within the time frame of 2–5 min as described above [47]. We therefore tested whether JA or other oxylipins may act as mobile signals as it has been described for systemin-induced long-distance signaling in tomato [5,6]. We used phenidone, an inhibitor of lipoxygenases, to manipulate JA accumulation. In a first test we applied phenidone to corn seedlings in a cut-stem assay overnight and then treated the plants with IE and analyzed local and distal responses. We found that phenidone significantly reduced JA accumulation in both segments (Figure 3B), making it an effective tool to manipulate JA biosynthesis. In a second assay we mixed IE and phenidone and applied the mixture to a damage site on intact plants. Controls were treated with only the IE at the same concentration. We found that local application of phenidone significantly reduced JA accumulation in this segment (Figure 3A [48]); however, we did not find any reduction in JA accumulation in the distal segment.

These results suggest that JA and also other oxylipins are not mobile signals and that other signaling mechanisms are likely to propagate the IE signal to distal parts of the leaf.

### 2.4. Alamethicin-Induced Signaling Leads to Distal Jasmonic Acid Accumulation

**Figure 4:**
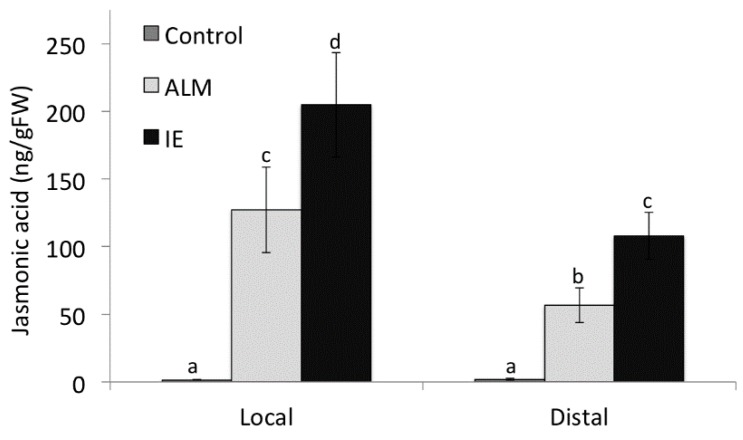
Comparison of insect elicitor (IE) and alamethicin (ALM) on local and distal jasmonic acid accumulation. Different letters above each bar indicate statistical difference determined by ANOVA analysis followed by Tukey tests where appropriate (*p* < 0.05). *N* = 4, error bars represent standard deviation.

In recent years, evidence has accumulated suggesting that electrical signaling is responsible for the activation of plant defense responses to insect herbivory in distant undamaged tissues of the same plant. For example, when *Spodoptera litoralis* feeds on bean plants a wave of plasma membrane depolarizations spreads to undamaged areas of the damaged leaves and activates defenses [49]. In a related study it was shown that systemic signaling may be propagated by so-called “system potentials”, which are thought to be regulated by the plasma membrane H^+^-ATPase [9]. In tomato, the use of ionophores further supported the notion that changes in membrane polarity are essential for the activation of plant defenses [11]. Further evidence for a similar kind of signaling came from using Alamethicin (ALM), a channel-forming peptide from the plant parasitic fungus *Trichoderma viride*, as an elicitor of plant defenses [50,51]. Local application of this elicitor caused long-distance signaling by means of membrane depolarization [52]. To test whether the application of ALM also causes long-distance signaling in corn, we compared the activities of ALM and IE by analyzing the local and distal response to these elicitors through the accumulation of JA. We found that ALM, like IE, did induced JA accumulation not only locally at the application site, but also in distal segments of the treated leaf (Figure 4).

ALM forms voltage-gated ion channel in membranes with a strong preference for protons and is therefore often used to mimic reactions that act through PM depolarization, which then propagates as a signal throughout the plant usually resulting in JA accumulation and the production of volatiles. This effect was shown for various plant species and is likely independent of receptors [50,51,52]. However, it also has to be stated that ALM does not only induce JA in plants, but also ethylene, salicylic acid, and abscisic acid [45]. In *Arabidopsis* current injections elicited the accumulation of JA-Ile and subsequent enrichment in RNAs encoding key jasmonate regulators [10]. A further screening revealed three glutamate receptor-like genes, which when knocked-out, significantly reduced long-distance signaling after wounding. However, as shown previously and above, only in the presence of IE do corn plants signal to distant parts of the same leaf resulting in JA accumulation, while no accumulation of JA has ever been reported in other systemic parts of the plant [12].

### 2.5. Priming by Green Leaf Volatiles Increases Distinct Free Fatty Acid in Different Plant Species

Distal signaling was shown previously to be characteristic for IE activity in corn leaves resulting in JA accumulation in those areas [12]. GLV-induced priming enhanced this effect of IE significantly, while MW-induced accumulation of JA is not affected. This specific link between GLV-induced priming and IE signaling appears to be at the core of how these two events are connected. Nonetheless, it is still unknown what causes this enhanced signaling by IE in distal parts of the leaf. In the past, we have shown GLV induce transcript accumulation for a wide array of defense genes covering transcriptional regulation, octadecanoid signaling, as well as direct and indirect defenses [53]. However, this transcriptional activity has been transient, lasting in most cases for only 2–3 h. Also, while GLV induce a transient JA accumulation in monocot plants, no such activity has ever been shown for dicot plants, although they too respond to GLV exposure by exhibiting a specific priming effect towards herbivory-related defenses [34,35,36,37,38]. Aside from having comparable priming effects on dicot and monocot plants and the induction of certain defense-related genes, no shared signaling compounds have been described to date that would explain these common responses. We therefore started a comprehensive metabolic analysis of different plant species and their response to GLV covering both monocot and dicot plants with the goal of identifying common elements that would help to explain the biological activities of GLV in the context of defense priming.

**Figure 5:**
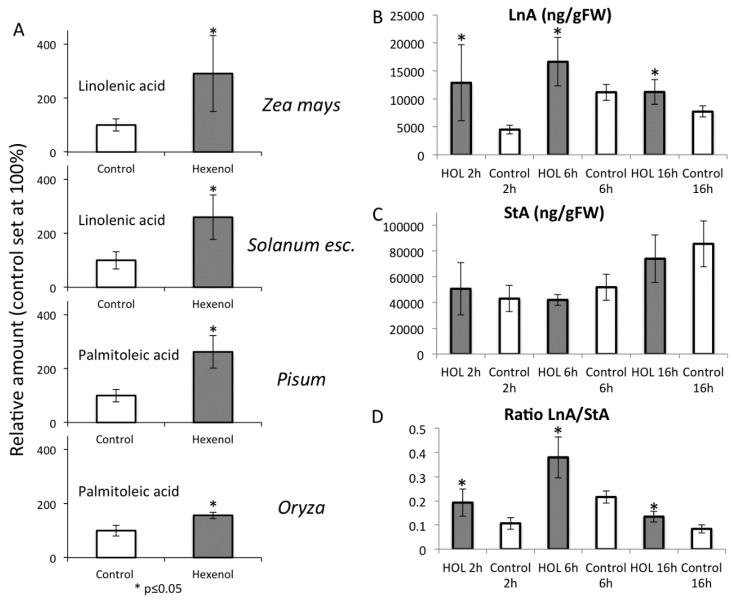
(**A**) Increases in free fatty acids in different plant species after exposure to *Z*-3-hexenol as our model GLV. Plants were exposed to 20 µg of *Z*-3-hexenol (HOL) for 15 min and free fatty acids analyzed by GC/MS. Note that *Zea* and *Solanum* show increased linolenic acid (LnA) levels, whereas in *Pisum* and *Oryza* palmitoleic acid was increased. Relative amounts are displayed with free fatty acid levels in control plants set at 100%. * denotes significant differences (*t-*test, *p* ≤ 0.05). (**B**–**D**) Effects of prolonged GLV treatments on free linolenic acid levels in maize leaves. Maize plants were exposed to 20 μg of GLV for the time indicated. (**B**) Amounts of free LnA after 2, 6, and 16 h; (**C**) Amounts of free stearic acid (StA) after 2, 6, and 16 h; (**D**) Ratios of LnA to StA after 2, 6, and 16 h. * denotes significant differences (*t-*test, *p* ≤ 0.05). *N* = 5, error bars represent standard deviation.

We exposed various plants including maize (*Zea mays*), peas (*Pisum sativum*), tomato (*Solanum leucopersicum*), and rice (*Oryza sativa*) to physiological concentrations of *Z*-3-hexenol (HOL), our model GLV. We found that all plants tested exhibited an increase in various unsaturated free fatty acid (fFA). For corn and tomato, we found a significant increase in α-linolenic acid (LnA), while in pea and rice palmitoleic acid increased. Interestingly, we found that these changes occur very quickly within the first 15 min of exposure to HOL (Figure 5A).

We further tested for the long-term effect of HOL on free fatty acid levels in maize by exposing seedlings to HOL for 2 h, 6 h, and 16 h (overnight). We found that over this time course, increased free LnA levels were maintained (Figure 5B). At the same time, stearic acid (StA) levels showed no difference between HOL-treated and control plants (Figure 5C); however, a minor shift in free StA levels was observed. We also observed that fFA levels vary significantly between individual plants and found that expressing changes of fFA levels in maize as ratios between LnA and StA is more accurate (Figure 5D).

### 2.6. Increased Free Linolenic Acid Levels Stimulate Insect Elicitor Induced Jasmonic Acid Accumulation

**Figure 6:**
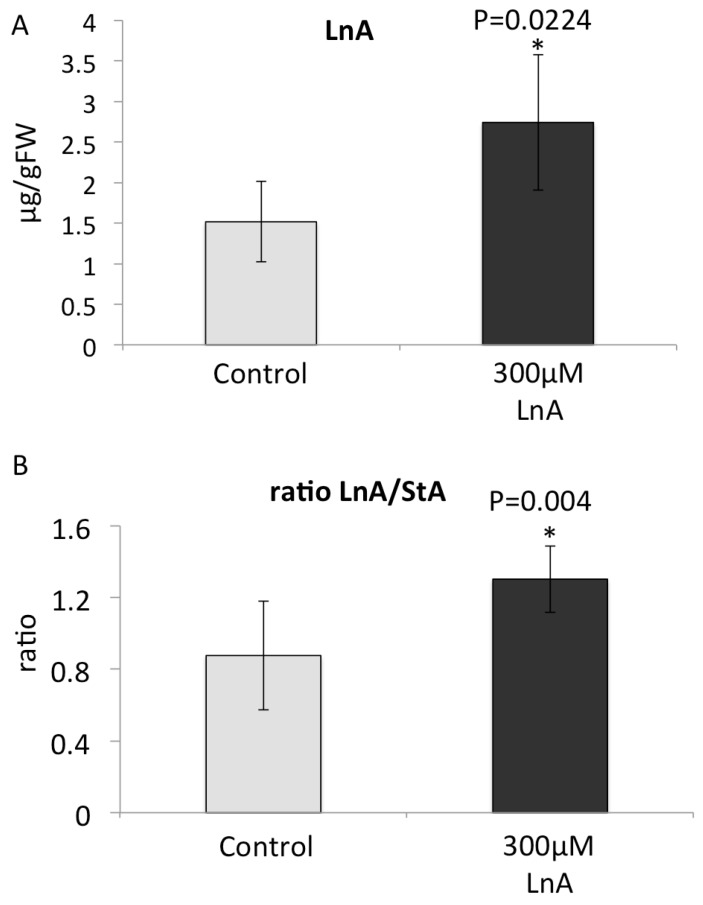
(**A**) Linolenic acid (LnA) accumulation after treatment with 300 µM LnA overnight; (**B**) Ratio of free LnA to free stearic acid (StA) after treatment with 300 µM LnA overnight. * denotes significant differences (*t-*test, *p* ≤ 0.05). *N* = 4, error bars represent standard deviation.

**Figure 7:**
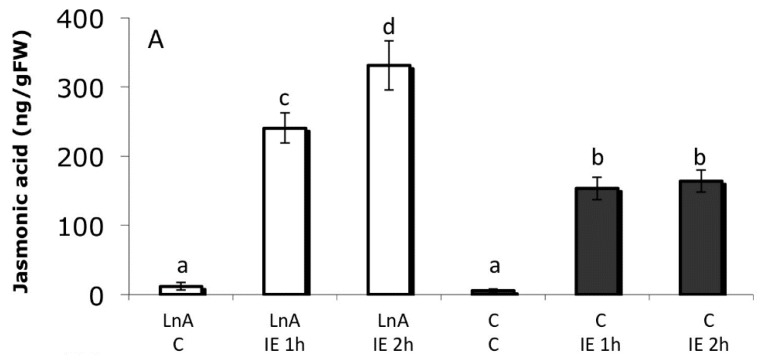

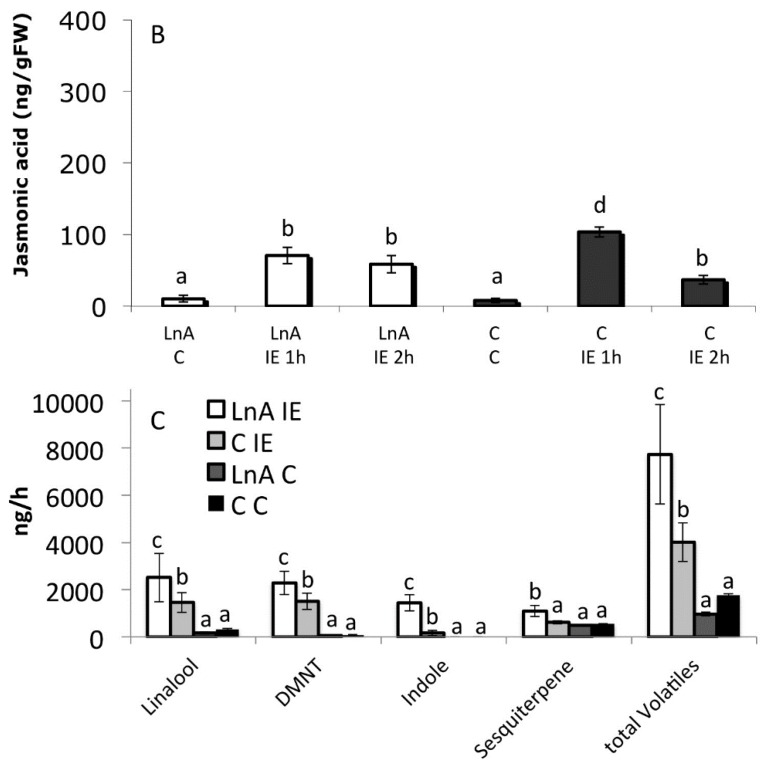
(**A**) Effects of free linolenic acid (LnA) on insect elicitor (IE) induced jasmonic acid accumulation; (**B**) Effects of free linolenic acid (LnA) on mechanical wounding (MW) induced jasmonic acid accumulation; (**C**) Comparison of volatiles release from corn seedlings pre-treated LnA and then with IE. Controls were treated similarly, but without LnA. Plant were treated with 300 μM aqueous solution of the respective fatty acid overnight and then treated by either MW or application of IE. JA was measured 1 and 2 h after treatment. JA was analyzed by GC/MS. Plant volatiles were collected between 4 and 5 h after treatment with IE [34]. Different letters above each bar indicate statistical difference determined by ANOVA analysis followed by Tukey tests where appropriate (*p* < 0.05). *N* = 5, error bars represent standard deviation. Statistical analysis was done for each volatile group independently.

To further explore the effects of fFA we treated corn seedlings with LnA at physiological concentrations that reflected the increase observed in the previous study after GLV exposure (Figure 5 and Figure 6). We found that treatment with 300 µM LnA overnight in a cut-stem assay produced similar LnA levels and LnA/StA ratios (Figure 6A,B). We then treated these plants either with IE or by MW and analyzed JA accumulation in the distal area of the treated leaf. As with GLV priming, we found that LnA treated plants accumulated more JA after IE treatment when compared to their respective control (Figure 7A). Similar to GLV-induced priming, we also found that MW alone did not result in an increased JA accumulation in LnA-treated plants (Figure 7B). As a consequence of this increase in JA accumulation in LnA-treated plants we also found a significant increase in volatile production (Figure 7C).

**Figure 8:**
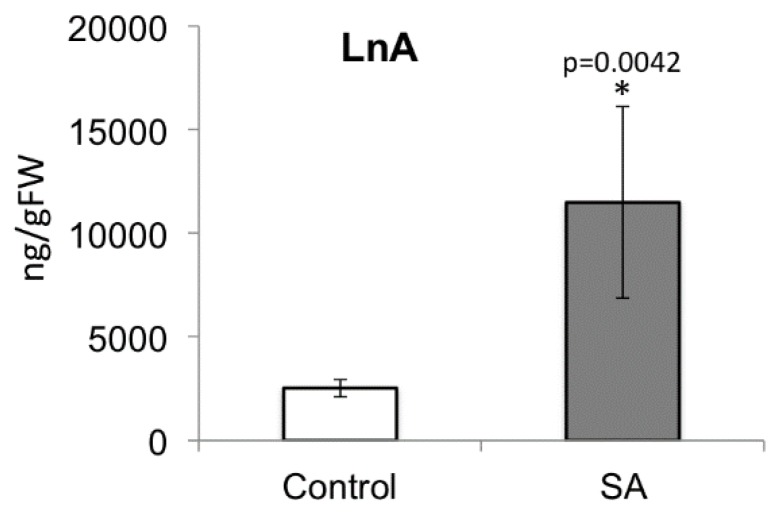
Free linolenic acid (LnA) levels in corn plants treated with salicylic acid (SA) overnight. Corn seedlings were treated overnight with 50 mL of a 100 μM salicylic acid solution added to the soil. * denotes significant differences (*t-*test, *p* ≤ 0.05). *N* = 4, error bars represent standard deviation.

Previously, we investigated the effects of salicylic acid (SA) on maize responses to MW and IE treatment [54]. In that study we found that overnight treatment with low concentrations of SA had the same priming effect on IE-induced responses (expressed as JA accumulation and volatile release) as described for corn seedling exposed to GLV, while the response to MW was not affected. In a supplemental study we found that this SA treatment of corn seedlings also increased the amount of free LnA (Figure 8).

These results clearly show that increased LnA levels have a priming effect on IE-induced responses similar to that observed after GLV treatment. Since LnA is the natural precursor for the biosynthesis of JA, we tested maize seedlings for the direct incorporation of LnA in JA after IE treatment. Maize seedlings were treated in a cut-stem assay with ^13^C-labelled LnA for 16 h overnight and then with IE. JA accumulation was tested for the incorporation of this labeled precursor; however, we found no evidence for externally applied free LnA being directly utilized for JA biosynthesis since no incorporation of ^13^C-labelled LnA into JA could be detected [55]. Also, MW of plants with elevated LnA levels did not cause any further JA accumulation when compared to control plants. It is therefore unlikely that fFA serve as a precursor for JA biosynthesis. We are currently testing other fatty acids including palmitoleic acid, linoleic acid, and γ-linolenic acid for their priming activity. First results indicate that they exhibit a similar activity to the one described above for LnA.

### 2.7. Decreased Free Linolenic Acid Levels Reduce Insect Elicitor-Induced Jasmonic Acid Accumulation

**Figure 9:**
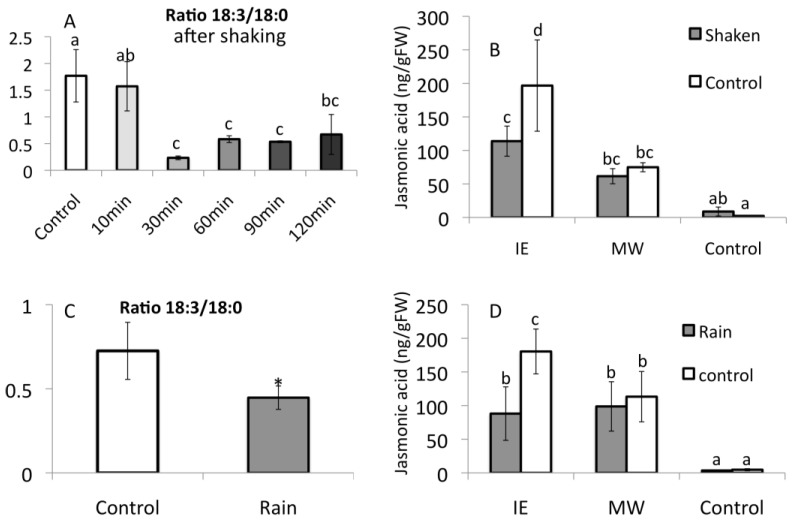
Effects of plant movements on free fatty acid levels and jasmonic acid accumulation. (**A**) Effect of shaking on free fatty acid levels in corn leaves. Plants were shaken for 15 s (upper panel); Free fatty acids were extracted and measured at times indicated in upper panel after treatment; (**B**) Effect of shaking on jasmonic acid accumulation after treatment with insect elicitor (IE, volicitin) or mechanical wounding (MW) for 1 h. Plants were shaken as above and then left standing for 30 min before treatment with IE or MW. (**C**) Effect of simulated rain on free fatty acid levels in corn leaves. Plants were treated with simulated rain for 30 s Free fatty acids were analyzed 30 min after rain treatment. Displayed are the ratios between linolenic acid (LnA) and stearic acid (StA). StA levels did not change upon treatments described herein. (**D**) Effects of rain on jasmonic acid accumulation after treatment with insect elicitor (IE) or mechanical wounding (MW) for 60 min. Different letters above each bar indicate statistical difference determined by ANOVA analysis followed by Tukey tests where appropriate (*p* < 0.05). * denotes significant differences (*t-*test, *p* ≤ 0.05). *N* = 6, error bars represent standard deviation.

To further support our theory that fFA levels have an IE-specific priming effect, we performed experiments that reduced LnA levels including shaking as a simulation of wind-induced movements as well as simulated rain by using a water can with a sprinkler spout. For both treatments we found that even after a short exposure to movement by shaking or simulated rain the free LnA levels were significantly reduced within 30 min (Figure 9A (shaking) and Figure 9C (rain)). Maize plants treated with IE or mechanical wounding at this time showed a significant reduction in the accumulation of JA after IE treatment (Figure 9B,D), but no significant changes with mechanical wounding.

Plants in their natural environment are constantly exposed to abiotic factors that cause movement including wind and rain. In some cases this mechanical stimulation can lead to altered growth responses, or thigmomorphogenesis, a process in which JA seems to play an important role [56,57]. In maize these abiotic factors have a significant and fast impact on fFA ratios with potential consequences for other responses including defense. However, we have never detected any significant impact of this treatment on growth behavior. The results also show that simple handling of plants may already affects fFA levels in a laboratory setting and can at least be partially responsible for strong variations in results.

Free FA and their derivatives have been described to play an important role in the regulation of many processes in animal and plant systems [58,59,60]. Traditionally, most of these regulatory functions were attributed to modified fatty acids, in particular those oxidized by either a lipoxygenases or a cyclooxygenase [61,62]. However, unmodified fFA are involved in a multitude of regulatory processes and many components of the fFA signaling events have been identified. For example, fFA regulate the expression of many genes, often those involved in nutrition-related processes, but also those related to inflammation, oxidative stress, apoptosis, and lipid homeostasis [63]. As can be seen from these examples, fFA in animals are involved in a multitude of physiological processes.

In plants, fFA and their derivatives have been described as signaling molecules in very few biological processes. Exogenous and endogenous unsaturated and poly-unsaturated fatty acids have been shown to alter gene expression thereby significantly influencing the outcome of plant-microbe and plant-insect interactions [64,65]. Increased levels of LnA have been described for wounding and interactions with insect herbivores and parasites [66,67]. Treatment of plants with high concentrations of LnA (2 mM) was shown to activate some defense responses [68]. Baudouin *et al.* [69] described the inhibitory effect of free fatty acids, in particular LnA, on a protein phosphatase involved in the MAP kinase pathway after wounding. In wheat (*Triticum aestivum*) a fungal-derived lipase triggered the release of fFA, which resulted in an inhibition of callose deposition [70,71]. In *Arabidopsis*, a defective stearoyl-acyl carrier protein desaturase, which converses stearic acid to oleic acid, caused a reduction of typical JA-regulated defense genes, thereby significantly reducing resistance to *Botrytis cinera*. On the other hand, this mutation caused the constitutive expression of PR genes, which enhanced the resistance to *Hyaloperonospora arabidopsidis* [72,73]. Likewise, a mutation in a particular fatty acid desaturase (FAD7) lead to the accumulation of salicylic acid, a major regulator of defenses against biotrophic pathogens, and enhanced resistance to aphids in a salicylate-dependent manner [74]. In addition, lipid transfer proteins, which are capable of exchanging lipids (and also fFA) between membranes, are often localized on the extracellular side of the plasma membrane, and have been implicated in the regulation of plant-pathogen defense responses [75].

Our results show fFA as a highly dynamic system that responds quickly to changes in the biotic and abiotic environment. In maize, fFA levels/ratios can change rapidly under certain biotic and abiotic stresses. While treatment with GLV increased free LnA levels, movement of plants reduced them. We have further shown that these altered free LnA levels correlate positively with IE-induced JA accumulation. Since fFA stimulate specifically distal IE-induced signaling, we postulate a role for fFA as mediators in this distant signaling process. In contrast, MW in maize is a very local event and JA accumulates only at the damage site, but not in distant tissues. Therefore, free LnA has no effect on MW-induced JA accumulation. Some fFA, in particular LnA, have been described to affect specific ion channels [76]. Also, LnA and other unsaturated fFA have been found to inhibit callose depositions on plasmodesmata by directly blocking callose synthase activity [77]. Since electrical signaling is very likely the cause of long distance signaling induced by IE, the stimulation of specific ion channels and the inhibition of plasmodesmata blocking through callose could play a role in this process by creating stronger signaling events after IE treatments resulting in increased JA accumulation. However, since we are just beginning to understand long distance signaling in plants, more efforts must be undertaken to study not only the potential role of fFA in the process, but also other factors that are as yet undetected.

## 3. Experimental Section

### 3.1. Chemicals

*Z*-3-hexenol, 3-octen-2-one, and dihydro-jasmonic acid-methyl ester were obtained from Bedoukian (Bedoukian Research, Danbury, CT, USA). Dihydro-jasmonic acid-methyl ester was converted to dihydro-jasmonic acid by alkaline hydrolysis and used as the internal standard for jasmonic acid. Linolenic acid was purchased from Sigma-Aldrich. All solvents used were analytical grade. Volicitin was generously provided by Dr. Hans Alborn (USDA, ARS, CMAVE, Gainesville, FL, USA).

### 3.2. Plant Material

*Zea mays* (var. Kandy King, J.W. Jung Seed Co., Randolf, WI, USA) plants were grown in soil (Redi Earth Plug and Seedling Mix, Sun Gro, Agawam, MA, USA) in a growth chamber with a 12 h photoperiod, 60% relative humidity at 26 °C for two to three weeks. Light intensity was set at app. 150 µmol m^2^·s^−1^. Plants used for experiments were at the V_2_ stage. Pea plants (*Pisum sativum*), tomatos (*Leucopersicum esculentum*), and rice (*Oryza sativa*) were grown from seeds under the same conditions as maize. 3–4 week-old plants were used for experiments.

### 3.3. Plant Treatments

To treat corn seedlings with GLV, intact maize plants were placed in 10 L glass cylinders. 10 μg of *Z*-3-hexenol (dissolved in dichloromethane, 1 μg/μL) was pipetted onto a cotton ball in the glass cylinder. Controls consisted of a plant in a chamber with 10 μL of pure dichloromethane applied onto a cotton ball. For short-term treatments plants including corn, pea, tomato, and rice were exposed to *Z*-3-hexenol for 15 min. The second leaf was then cut and shock frozen in liq. N_2_. Corn seedlings were also exposed for 2 h, 6 h, and 16 h to analyze for long-term effects of GLV on free fatty acid levels. For long-term priming experiments corn plants were exposed to these chemicals overnight (15 h) and the second leaf of each seedling was harvested and immediately frozen in liquid N_2_ for further processing. For induction with IE (here: volicitin (*N*-(17-hydroxy-linolenoyl)-glutamine)) an area of about 2 mm × 10 mm on the second leaf of intact maize plants was scratched with a razor blade and 10 μL of IE (100 pmol·μL^−1^, in 50 mM KPi buffer, pH 8) were immediately added to the wounded site. For wounding, plants were treated similarly, but without the addition of IE. Segments of about 2.5 cm were taken from the wounded site (local) and distal (Figure 1) and immediately shock-frozen in liquid N_2_. These leaf segments were then analyzed for JA accumulation. To estimate the signaling speed of IE with regard to JA accumulation in the distal part of the leaf we first treated leaves of corn seedlings with IE as described above. Controls were treated with MW alone. After 2 min, 5 min, 10 min, and 30 min, respectively, treated leaves were cut 1 cm above the damage site and placed in water. After a total of 45 min including the initial treatment time before the first cut leaves were taken out of the water and the first centimeter close to the previous cutting site was again remove, while the next 3 cm of the leaf comprising the distal part were shock-frozen in liq. N_2_ for JA analysis.

Alamethicin (ALM) was dissolved in methanol at 1 mg·mL^−1^ and then diluted with buffer (50 mM KPi, pH 8) to a final concentration of 10 μg·mL^−1^. From this 10 μL were applied to a damage site on the maize leaf as described above for IE. After 60 min a local segment comprising the damage site and a distal segment was taken and shock-frozen in liq. N_2_ for JA analysis.

#### 3.3.1. Treatment of Corn Seedlings with Free α-Linolenic Acid

For treatment of corn seedlings with free fatty acids (fFA), plants were cut at the mesocotyl and immediately placed in a solution containing 300 μM α-linolenic acid (LnA) in water/methanol (100:1 *v*/*v*). Plants were allowed to take up LnA overnight (16 h). The next morning plants were treated with IE or MW as described above for 60 min and 120 min. Leaf segments (5 cm) comprising the local IE-application site (local) and distal areas of the same leaf (distal) were taken and immediately shock-frozen in liq. N_2_ for subsequent JA and LnA analysis as described below. Segments weigh between 60 and 120 mg and are well within the range for effective plant hormone extraction.

#### 3.3.2. Pharmacological Effects of Phenidone on JA Accumulation

To study the pharmacological effects of phenidone on JA accumulation in maize seedlings that were treated with IE, plants were cut at their base and immediately transferred into vials with either 2 mM phenidone in water or water as a control. After overnight incubation plants were treated the next day with IE for 60 min. Leaf segments comprising the local and distal area were analyzed for JA accumulation.

To test phenidone for its effects on local JA production and mobilization of JA from the IE-application site we mixed a 2 mM phenidone solution with IE 1:1 and applied the mixture to the damage site. Controls were treated with IE only at a similar concentration. One hour after treatment, segments comprising the local and distal area of the leaf were then analyzed for JA accumulation.

#### 3.3.3. Effects of Movement on Free Fatty Acids and JA Accumulation

To test for the effects of movement on fFA levels we exposed corn seedling to either shaking or by simulated rain. For shaking plants were placed on a laboratory shaker at 90 rpm for 15 s at an approximate movement radius of 5 cm. After this, plants were allowed to sit without any further movement for 10, 30, 60, 90, and 120 min before the two youngest leaves were cut and immediately shock-frozen in liq. N_2_ for subsequent fFA analysis. After establishing time points with low fFA levels, we used these time points for further experiments including rain simulation. Unmoved plants were watered with a water can with a sprinkler spout from about 1 m high for 30 s Control plants were watered from below to allow for similar water content of the soil. After 30 min the two youngest leaves were cut and immediately shock-frozen in liq. N_2_ for fFA analysis. To test for the effects of movement on plant responses to IE, we applied movement to corn seedlings as described above and plants were allowed to rest for 30 min. Then, plants were treated with IE as described above and leaf segments including the IE-application site were cut and immediately shock-frozen in liq. N_2_ for JA analysis.

### 3.4. Quantification of Jasmonic Acid and Free Fatty Acids

Extraction and quantification was performed as described previously [19,34,45]. In brief, about 100 mg of plant material were frozen in liquid N_2_, and homogenized in acidic propanol/water in a Precellys tissue homogenizer (MO BIO Laboratories, Carlsbad, CA, USA) at 6000 shakes per minute. After adding 1 mL dichloromethane the organic phase was taken, dried down, and methylated with diazomethane. Methylated and thus volatile compounds were then separated from the complex mixture by vapor phase extraction and analyzed by CI-GC/MS. Quantification was based on the internal standard and the fresh weight of the plant material.

### 3.5. Statistical Analysis

Data are presented as means ± SD of three to six replicates, unless otherwise specified. Data were analyzed for significance with *t*-test (*p* < 0.05). ANOVAs were performed on concentrations of JA, free fatty acids, and induced VOC. Significant treatment effects were investigated when the main effects of the ANOVAs were significant (*p* < 0.05). Where appropriate, Tukey tests were used to correct for multiple comparisons between control and treatment groups. Before statistical analysis, all data were subjected to square root transformation to compensate for elevated variation associated with larger mean values.

## 4. Conclusions

Distant signaling induced by IE is one of the major targets for GLV-activated defense priming in maize. In this context, fFA appear to play a significant role by mediating this signaling process, resulting in altered defense response intensity. While high fFA levels increase the IE-induced defense response, low fFA levels cause a decrease. Biological activities related to plant defense have been described before for various fatty acids, but no correlation between altered fFA levels caused by different stresses and the intensity of defense signaling has been described. However, while in maize changes in LnA levels were mainly observed, other plants showed changes in other fFA (e.g., PeicA) after these treatments. It is therefore unclear how specific this effect is with regard to individual fFA. This is currently under investigation.
